# 

**Published:** 1907-03-02

**Authors:** 


### POPULAR NURSING TEXT-BOOKS.

The works contained in this Series are well written by expert teachers of the subjects of which they treat, and will form a very valuable and useful addition to a Nurse's Library. Each volume is strongly bound in cloth cover, and contains about 100 pages.

Price 1/- each volume, post free.

**PRACTICAL HINTS ON DISTRICT NURSING.**
By Miss Amy Hughes.

**HOSPITAL AND PRIVATE NURSING.**
By Miss S. E. Orme.

**THE MIDWIVES' POCKET BOOK.**
With Prefatory Chapter on the Midwives Bill.
By Miss Honnor Morten, Author of "The Nurses' Dictionary," &c. &c.

**FEVERS AND INFECTIOUS DISEASES: Their Nursing and Practical Management.**
By William Harding, M.D.Ed., M.R.C.P. Lond., Author of "Mental Nursing," &c.

**THE CARE OF CONSUMPTIVES.**
By W. H. Daw, M.R.C.S., L.R.C.P.Lond.

**NOTES ON PHARMACY AND DISPENSING FOR NURSES.**
New and Revised Edition.
By C. J. S. Thompson.

**MENTAL NURSING.**
By William Harding, M.B.Ed., M.R.C.P. Lond.

**SICK NURSING AT HOME.**
By Miss L. G. Moberly.

**GYNÆCOLOGICAL NURSING.**
By G. A. Hawkins-Ambler, F.R.C.S., Author of "The Commonwealth of the Body."

**SYLLABUS OF LECTURES TO NURSES.**
By Andrew Davidson, M.D.

**The Scientific Press, Ltd.,**

☛ *Catalogue of Nursing Manuals sent post free on application.* ☚

**Publishers of Nursing Text-Books, Charts, and Case-Books of all Descriptions.**

**28 & 29 SOUTHAMPTON STREET, STRAND, LONDON, W.C.**

